# Recombinant Protein Expression and Purification of N, S1, and RBD of SARS-CoV-2 from Mammalian Cells and Their Potential Applications

**DOI:** 10.3390/diagnostics11101808

**Published:** 2021-09-30

**Authors:** Julio García-Cordero, Juvenal Mendoza-Ramírez, David Fernández-Benavides, Daniela Roa-Velazquez, Jessica Filisola-Villaseñor, Sandra Paola Martínez-Frías, Erik Saul Sanchez-Salguero, Carlos E. Miguel-Rodríguez, Jose L. Maravillas Montero, Jose J. Torres-Ruiz, Diana Gómez-Martín, Leopoldo Santos Argumedo, Edgar Morales-Ríos, Juan M. Alvarado-Orozco, Leticia Cedillo-Barrón

**Affiliations:** 1 Departamento de Biomedicina Molecular CINVESTAV IPN, Av. IPN # 2508 Col, San Pedro Zacatenco, México City 07360, Mexico; jugarcia@cinvestav.mx (J.G.-C.); noe.mendoza@cinvestav.mx (J.M.-R.); paola.martinez@cinvestav.mx (S.P.M.-F.); erick.sanchez@cinvestav.mx (E.S.S.-S.); carlos.miguel@cinvestav.mx (C.E.M.-R.); lesantos@cinvestav.mx (L.S.A.); 2 Centro de Ingeniería y Desarrollo Industrial (CIDESI), Av. Playa Pie de la Cuesta No. 702, Desarrollo San Pablo, Querétaro 76125, Mexico; david.fernandez@cidesi.edu.mx (D.F.-B.); juan.alvarado@cidesi.edu.mx (J.M.A.-O.); 3 Departamento de Bioquímica CINVESTAV IPN, Av. IPN # 2508 Col, San Pedro Zacatenco, México City 07360, Mexico; daniela.roa@cinvestav.mx (D.R.-V.); jessica.filisola@cinvestav.mx (J.F.-V.); edgar.morales@cinvestav.mx (E.M.-R.); 4 Red de Apoyo a la Investigación, Universidad Nacional Autónoma de México e Instituto Nacional de Ciencias Médicas y Nutrición Salvador Zubirán, Vasco de Quiroga 15, Tlalpan, México City 14080, Mexico; maravillas@cic.unam.mx; 5 Departamento de Inmunología y Reumatología, Instituto Nacional de Ciencias Médicas y Nutrición Salvador Zubirán, Vasco de Quiroga 15, Tlalpan, México City 14080, Mexico; jiram_torres@hotmail.com (J.J.T.-R.); d_gomar@hotmail.com (D.G.-M.)

**Keywords:** severe acute respiratory syndrome coronavirus 2, coronavirus disease, spike protein, nucleocapsid, receptor binding domain

## Abstract

The coronavirus disease 2019 (COVID-19) pandemic has reached an unprecedented level. There is a strong demand for diagnostic and serological supplies worldwide, making it necessary for countries to establish their own technologies to produce high-quality biomolecules. The two main viral antigens used for the diagnostics for severe acute respiratory syndrome coronavirus (SARS-CoV-2) are the structural proteins spike (S) protein and nucleocapsid (N) protein. The spike protein of SARS-CoV-2 is cleaved into S1 and S2, in which the S1 subunit has the receptor-binding domain (RBD), which induces the production of neutralizing antibodies, whereas nucleocapsid is an ideal target for viral antigen-based detection. In this study, we designed plasmids, pcDNA3.1/S1 and pcDNA3.1/N, and optimized their expression of the recombinant S1 and N proteins from SARS-CoV-2 in a mammalian system. The RBD was used as a control. The antigens were successfully purified from Expi293 cells, with high yields of the S1, N, and RBD proteins. The immunogenic abilities of these proteins were demonstrated in a mouse model. Further, enzyme-linked immunosorbent assays with human serum samples showed that the SARS-CoV-2 antigens are a suitable alternative for serological assays to identify patients infected with COVID-19.

## 1. Introduction

Severe acute respiratory syndrome coronavirus 2 (SARS-CoV-2) is a recently identified virus that is responsible for the current pandemic of coronavirus disease 2019 (COVID-19), which has globally infected and killed millions of people and has seriously damaged the world economy [1,2]. Patients with COVID-19 can be asymptomatic or symptomatic. The most common symptoms include fever, dry cough, sneezing, headache, and sore throat, as well as general malaise, fatigue, and shortness of breath [3]. The incubation period of SARS-CoV-2 is between 7 and 14 days, during which it is potentially contagious. Patients who progress to severe forms of the disease present respiratory disorders, such as bilateral pneumonia (75–98%), with their chest X-ray images showing multiple shadows, and some patients exhibit interstitial infiltration associated with pneumonia. In accordance with recent findings, some patients present ataxia or seizures, suggesting the neurotropic impact of the viral infection [2,3,4,5].

SARS-CoV-2 belongs to the betacoronavirus family and is an enveloped virus approximately 80–120 nm in size. Its genome consists of a positive single-stranded RNA of approximately 30 kb in length. The SARS-CoV-2 viral particle is composed of four structural proteins, namely the spike (S), membrane (M), envelope (E), and nucleocapsid (N) proteins, as well as the viral RNA codes for nonstructural and accessory proteins [6,7,8]. The S protein is distributed in spike-shaped structures on the virus surface. The M protein has three transmembrane domains that give the virions its shape, promote membrane curvature, and bind to the N protein. The N protein contains two domains that can bind to the RNA genome. Finally, the E protein participates in virus assembly [9,10].

The S protein is an essential polyfunctional molecule involved in membrane fusion and viral entry and is highly immunogenic. Hence, it is the main antigen that should be targeted by vaccines and is used in diagnosis [10]. The S protein is a representative of class I viral fusion proteins. It is highly glycosylated and associates itself as homotrimers to form the characteristic spikes on different coronaviruses. The S protein is cleaved by host proteases, such as furin and trypsin transmembrane serin protease 2 (TMPRSS), to trigger its activation. Each monomer of the S protein is organized into two domains, designated as S1 and S2. The S1 fragment is in the N-terminal domain and contains the receptor-binding domain (RBD), which is crucial for tropism of the virus for the angiotensin-converting enzyme 2 (ACE2) receptor [10,11]. S2 is in the C-terminal region of the Spike protein, possessing a transmembrane domain and a fusion peptide fragment, which is essential for viral entry [12,13].

The nucleocapsid (N) is the most abundant protein found during SARS-CoV-2 infection, with its peak expression occurring during the first 12 h of infection. The N protein is composed of many polar amino acids and binds to the viral RNA through two domains. The N protein is a polyfunctional molecule, and one of its main functions is to protect the genome by forming ribonucleoprotein complexes, as well as to regulate viral RNA transcription during replication, stimulating the synthesis of viral proteins by interacting with the RNA genome via its two RBDs in the N- and C-terminal domains [14]. The SARS-CoV-2 N protein shows high homology with the SARS-CoV N protein, with a sequence identity of 90.52% [15]. Early studies of SARS-CoV showed that the antibody response in patients with SARS was directed predominantly at the nucleocapsid, showing high sensitivity and long persistence. The spike protein was the next most frequently targeted [16,17,18]. In agreement with the above data, recent studies of plasma or serum antibodies from patients with COVID-19 showed that antibodies for the nucleocapsid protein of SARS-CoV-2 are more sensitive compared to spike protein antibodies for detecting early infection [19].

Additionally, a longitudinal study using sera from patients who had recovered from COVID-19 showed IgG, IgA, and IgM antibodies against the N antigen [20] The IgM against S and IgG against N responses increase rapidly, while at six months, specific IgM against S and N proteins was undetectable. In contrast, IgG-S/N titers showed high levels at 6 months. These data confirm that the S, RBD, and N proteins are powerful antigens for COVID-19 antibody screening and disease diagnosis [20]. Interestingly, the N protein of most coronaviruses is highly immunogenic and, thus, may be a complementary antigen that can indicate the presence of an infection [19,21].

COVID-19 continues to be a health concern requiring urgent attention, as the pandemic has not been controlled. Furthermore, it is necessary for each country to have supplies for biological, diagnostic, and serological studies to carry out surveillance [22]. In this study, we designed and evaluated an expression system for the S1 and N proteins and estimated the level of purification of two recombinant proteins. Our results should be useful for performing surveillance studies.

## 2. Materials and Methods

### 2.1. Ethics Statement

#### 2.1.1. Mice

Six- to eight-weeks-old female BALB/c mice (H-2d) were housed in groups of 5 on the breeding facilities of the Centro de Investigacion y Estudios Avanzados del Instituto Politecnico Nacional (CINVESTAV). All animals were handled in accordance with institutional guidelines. The protocol procedures were approved by Animal Use Ethical committee 118.

#### 2.1.2. Patient Samples

The serum samples were collected as part of studies approved by the institutional review board of the Ethics Committee (COMBIOETICA-09-CEI-011-20160627) at the Instituto Nacional de Ciencias Medicas y Nutricion Salvador Zubiran. Blood samples were collected from patients 5–7 days after the onset of symptoms and RT-PCR confirmed COVID-19. Additionally, healthy serum samples collected before the COVID19 outbreak (2015, called pre-pandemic serum) for other studies were included. Both groups signed informed consent.

### 2.2. Cells and Viruses

#### 2.2.1. Expi 293F Cells

Expi293F cells (Thermo Fisher Cat#A14527) were grown in Expi293 Expression Medium (Thermo Fisher Cat#A1435101). This medium is chemically defined, serum-free, ready to use, and does not require any additional supplement. It was used in sterile TC flasks, vented, with a baffled bottom (Fisher Scientific Cat# BBV12-5) in an Orbital Shaker at 110 RPM (orbital diameter of 50 mm) at 37 °C and 5% CO_2_.

#### 2.2.2. Vero Cells

Vero cell lines were cultured in RPMI medium (pH 7.8; Gibco) supplemented with 5% FBS, 2 mM of l-glutamine, 1X NEAA, and 1X vitamins and incubated in a CO_2_ atmosphere at 37 °C.

### 2.3. Gene Syntheses and Plasmid Construction

The sequences coding for S1 (nucleotides 21563–23738) and N (nucleotides 28274–29533) proteins were based on GenBank (MN908947.3) [23]. Both sequences were synthesized along with the nucleotides encoding the signal peptide of the Spike sequence (first 45 nucleotides). Additionally, a hexahistidine (6xHis) encoding sequence tag at the C terminus was added. The synthetized nucleotides were cloned into the mammalian expression vector pcDNA3.1 (Invitrogen, Carlsbad, CA, USA) under the cytomegalovirus (CMV) promotor according to standard molecular techniques and were optimized for the expression in HEK 293 cells. The constructs were synthesized commercially (Gene Script, Piscataway, NJ, USA). The recombinant plasmids were designated as pcDNA3.1/S1 and pcDNA3.1/N. The spike S1 (721 aa) domain includes the modified polybasic cleavage site (RRAR to A), as described by Amanat and Krammer (2020). The N sequence was 420 aa long.

The plasmid encoding for the Spike RBD was kindly provided by Prof. Florian Krammer (Icahn School of Medicine at Mount Sinai, NY, USA).

### 2.4. Transfection of Expi293F Cells with SARS-2CoV2 Constructs

The plasmids pcDNA3.1/S1 and pcDNA3.1/N were transiently transfected into Expi293F cells (Thermo Fisher, Waltham, MA, USA). The cellular density was adjusted to 3 × 10^6^ cells/mL, in a final volume of 100 mL of Expi293 expression media in 500 mL baffled bottom flasks (Fisher Scientific Cat# BBV12-5) (Gibco, Thermo Fisher, Waltham, MA, USA), and was diluted in 3 mL of Opti-MEM medium. Next, the reagents of both tubes were mixed and incubated for 20 min at room temperature. After this time, the mixture was added to the cell culture. After 24 h of incubation, 300 µL of transfection enhancer-1 and 3 mL of transfection enhancer-2 were added and then incubated under shaking conditions. Finally, the supernatants were analyzed after 3 and 5 days post-induction, and the cultures were centrifuged at 4000× *g* for 20 min. The supernatant was collected in a Falcon tube and stored at 4 °C until the protein purification process, with 1× protease inhibitors added (SIGMA Cat# S8830-20) to prevent degradation.

### 2.5. Protein Production and Purification

On days 5 and 7 post-transfection, the transfected cells with pcDNA3.1/S1 and pcDNA3.1/N were centrifuged at 4000× *g* for 15 min and the supernatant was collected. The supernatants were concentrated to a final volume of 5 mL in a membrane with a cutoff of 50 kDa (Amicon^®^ Ultra Centrifugal filters, Darmstadt, Germany) for the RBD and N proteins, and a cutoff of 100 kDa for the S1 protein. The concentrated supernatants were dialyzed with buffer A, consisting of 25 mM of Na_2_HPO_4_, 25 mM of NaH_2_PO_4_, 500 mM of NaCl, and 5 mM of imidazole, to a final volume of 50 mL. Subsequently, protein purification was carried out by fast protein liquid chromatography (FPLC) (AKTA-Pure 25M, Cytiva, Uppsala, Sweden). The concentrated supernatant was injected into a His Trap HP 5 mL column (Cytiva, Marlborough, MA, USA) equilibrated with 10 volumes of Buffer A. The column was washed with 10 volumes of buffer A to remove any contaminants. Subsequently, elution was carried out using a concentration gradient with Buffer B, composed of 25 mM of Na_2_HPO_4_, 25 mM of NaH_2_PO_4_, 500 mM of NaCl, and 500 mM of imidazole. The pH of buffers A and B was different for each protein: for S1, the pH was 6.4; for RBD, the pH was 7.4; and for N, the pH was 9. Finally, the obtained fractions were analyzed by sodium dodecyl polyacrylamide gel electrophoresis (SDS-PAGE). The fractions containing the protein of interest were concentrated to 1 mL in a membrane (Amicon^®^ Ultra Centrifugal filters, Uppsala, Sweden) by centrifugation and subjected to FPLC purification using a size-exclusion column (a Superdex 75 Increase, Cytiva for both the RBD and N proteins, and a Superdex 200 Increase, Cytiva for S1) [24]. Initially, the column was equilibrated with 10 volumes of phosphate-buffered saline (PBS), and the protein was then injected, before the eluted fractions were recovered. Finally, the purity of the fraction obtained was analyzed by SDS-PAGE, and quantification was carried out using the Bradford method.

### 2.6. ELISA Assay

For ELISA assays, Greiner BioONE Microlon plates (655061; Fisher Scientific, Carlsbad, CA, USA) were coated overnight at 4 °C with S1, N, and RBD proteins diluted in PBS 1X pH 7.4. Plates were washed 3X with PBS containing 0.1% Tween 20 and blocked with PBS containing 3% (wt/vol) skim milk for 1 h. Next, serum was serially diluted in PBS with 1% (wt/vol) skim milk and added to the plates for 2 h. Peroxidase-conjugated anti-human IgM and IgG antibodies (627520; Invitrogen, 97225; Abcam respectively) were added. After three washes, the substrate *o*-phenylenediamine (P9029; Sigma-Aldrich) was added, and reaction was allowed for 20 min. The plates were read at 450 nm.

### 2.7. Immunofluorescence Analysis

Vero cells were seeded on glass cover slips (6 × 10^4^) (Bellco, Vineland, NJ, USA) contained in 24-well plates. As described before [25] The next day, monolayers were transfected with pcDNA 3.1/S1, pcDNA 3.1/N, and pCAGGS/RBD or transfected with the parental plasmid. Then, cells were incubated at 37 °C, and at 24 h post-infection, the cells were fixed with 4% paraformaldehyde (Sigma-Aldrich, St. Louis, MO, USA) in PBS for 20 min at room temperature, and then permeabilized with a solution of 0.1% Triton-X100 in PBS and blocked with 10% normal goat serum. The cell monolayer was incubated for 60 min with serum samples from patients with COVID-19; after washing, the corresponding fluorochrome-conjugated secondary antibody goat anti-human IgG-Biotin (Jackson Immunoresearch, Baltimore, MD, USA, Cat 109-066-088) was added and incubated for 1 h, followed by PBS washing and then FITC-Streptavidin (Invitrogen, CA, USA, Cat. 43-4311). Finally, the nuclei were labeled with DAPI (1 μg/mL) in PBS for 10 min, and the slides were mounted with Vectashield (Vector Labs, Burlingame, CA, USA). The images were captured using two different confocal microscopes (Leica SP2 and OLYMPUS FVX).

### 2.8. SDS-PAGE and Immunoblotting

Supernatant samples were resolved by SDS-PAGE using 12% or 15% gels for 80 min at 100 V (Mini-Protean Cell; Amersham Biosciences, Piscataway, NJ, USA), and further electrotransferred (120 V for 2 h) onto PDV (Hybond ECL; GE Healthcare, Little Chalfont, UK). Membranes were blocked with semi-skimmed Milk/PBS-Tween-20 and then incubated with the appropriate primary antibody such as anti-His, pre-pandemic serum samples, or monoclonal anti-S1, followed by the appropriate horseradish peroxidase (HRP)-conjugated secondary antibody (1:3000) in PBS-Tween-20. After further washing with PBS-Tween-20, the membranes were developed with western lightning-enhanced chemiluminescence reagent (Pearce, Rockford, IL, USA).

### 2.9. Immunogenicity Assay

To perform an in vivo immunization assay, four groups of five female BALB/c mice six- to eight-weeks-old were inoculated intraperitoneally 3 times with 10 μg of S1, N, and RBD recombinant proteins or PBS separately mixed with aluminum hydroxide (Alum) at intervals of 21 days. Blood samples were obtained at day 0 before the first doses and 20 days after each immunization (days 0, 20, 40, and 60). Samples were stored at −20 °C and then evaluated by enzyme-linked immunosorbent assay (ELISA).

## 3. Results

### 3.1. Construction of S1 and N Recombinant Plasmids

**Figure 1:**
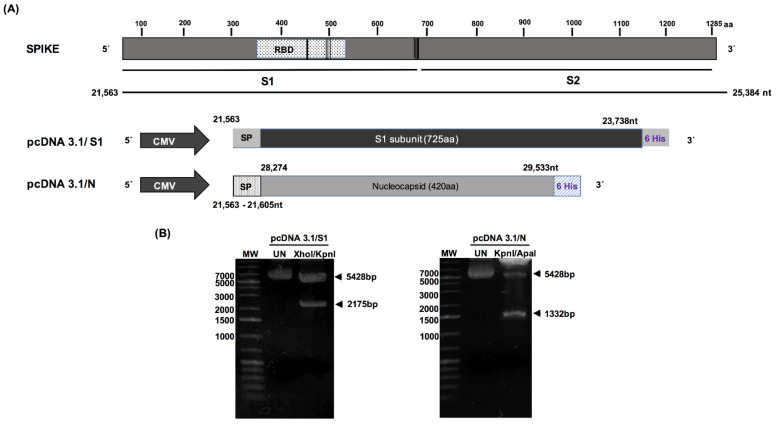
Severe acute respiratory syndrome coronavirus 2 protein constructs. (**A**) Schematic representations of the primary structure of full-length sequence of the spike protein (S) and schematic representation of constructs, namely pcDNA3.1/S1 and pcDNA3.1/N, with a 6XHis tag at the C-terminal and spike signal sequence at the N-terminal, which is flanked by XhoI and XbaI. (**B**) Enzymatic digestion of the synthesized recombinant plasmids, which were analyzed on a 1.2% (w/v) agarose gel. Lane 1, 5000 bp DNA size marker; lane 2, undigested plasmids; lane 3, enzymatic digestion of pcDNA3.1/S1 and pcDNA3.1/N with Xho and KpnI.

The entire sequences of the S1 domain, encoding 725 amino acids, and the N sequence, encoding 420 amino acids, were synthesized, and inserted into the pcDNA3 plasmid. Both sequences were placed under the CMV promoter, and the resultant proteins were labeled with a 6XHis tag at the C-terminus. Figure 1A shows a schematic representation of each plasmid with its specific inserted sequence. The constructs were used to transform *Escherichia coli* DH5 cells to produce plasmids for transfection. Following transformation with pcDNA3.1/S1 and pcDNA3.1/N, a recombinant clone for each plasmid was randomly selected for plasmid purification. The recombinant plasmids were purified from the bacterial cells using an Endo Free Plasmid Kit (Qiagen, Hilden, Germany). The purified plasmids were subjected to restriction enzyme digestion with XhoI/KpnI and KpnI/ApaI to confirm the presence of S1 and N-encoding inserts into the clones (Figure 1B). In both cases, restriction analysis confirmed the presence of an S1-encoding insert of 2175 bp and an N-encoding insert of 1332 bp. The resultant plasmids were sequenced, and all elements of the cassettes were confirmed to be in the same open reading frame. Additionally, we evaluated a previously published RBD construct as a control [24].

### 3.2. Expression of Recombinant S1 and N Proteins in Mammalian Cells

**Figure 2:**
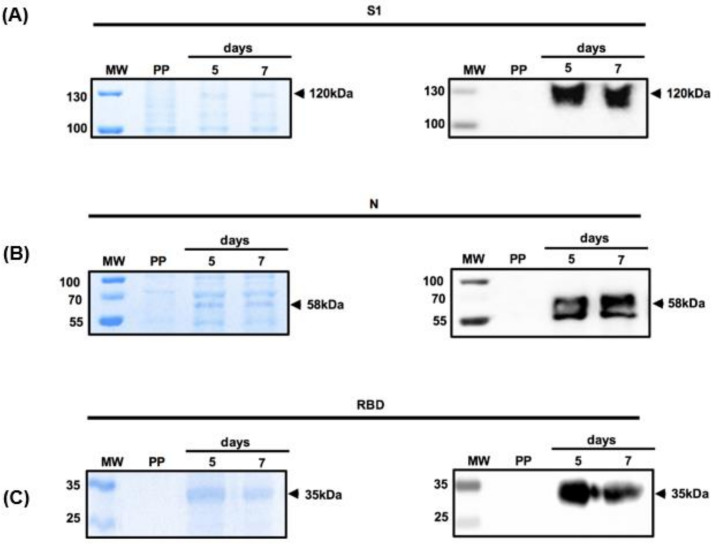
In vitro expression of the S1 domain of the spike protein, the nucleocapsid protein, and the receptor-binding domain in HEK293 cells. The recombinant proteins (**A**) S1, (**B**) N, and (**C**) RBD were subjected to SDS-PAGE and Western blot at day 5 and 7 after transfection. Lane 1, molecular weight marker (MW); lane 2, supernatant of cells transfected with parental plasmid (PP); lane 3, supernatant of cells obtained on day 5 after transfection; and lane 4, supernatant of cells obtained on day 7 transfection. The left of each panel shows the Coomassie blue staining, while the right shows the corresponding Western blot detected with anti-His antibodies.

To evaluate the expression of S1 and N proteins driven by the CMV promoter, human embryonic kidney (HEK)-293T cells were transiently transfected with the corresponding plasmids on days 5 and 7 after transfection, the cells were centrifuged, and the resultant supernatant was subjected to SDS-PAGE. Protein expression was confirmed by Western blot analysis using an anti-His antibody. The expression system produced high yields of both proteins (Figure 2). A protein band at 120 kDa in the Coomassie blue-stained SDS-PAGE suggested the expression of the S1 protein (Figure 2A), although the molecular weight was higher than predicted likely because of its glycosylation, which has been previously reported [26]. These data were confirmed by the Western blot analysis of transfected cells with pcDNA3.1/S1 using an anti-His antibody. Once again, although the predicted molecular weight was 80 kDa, we observed a protein band at 120 kDa (Figure 2A).

The analysis of the supernatant of transfected cells with pcDNA3.1/N showing a protein band at 57 kDa in the Coomassie blue-stained gel indicated the expression of the N protein, which was confirmed by Western blotting using an anti-His antibody. The molecular weight of the N protein was consistent with the predicted sizes of the recombinant proteins (Figure 2B). We also subjected the supernatant of RBD-transfected cells to protein purification, with a band observed at 37 kDa, as reported previously (Figure 2C). Importantly, all three recombinant proteins were soluble and secreted into the medium. Cells transfected with the parental plasmid showed no reactivity with the anti-His antibody.

### 3.3. Optimization of the Protein Purification Process

**Figure 3:**
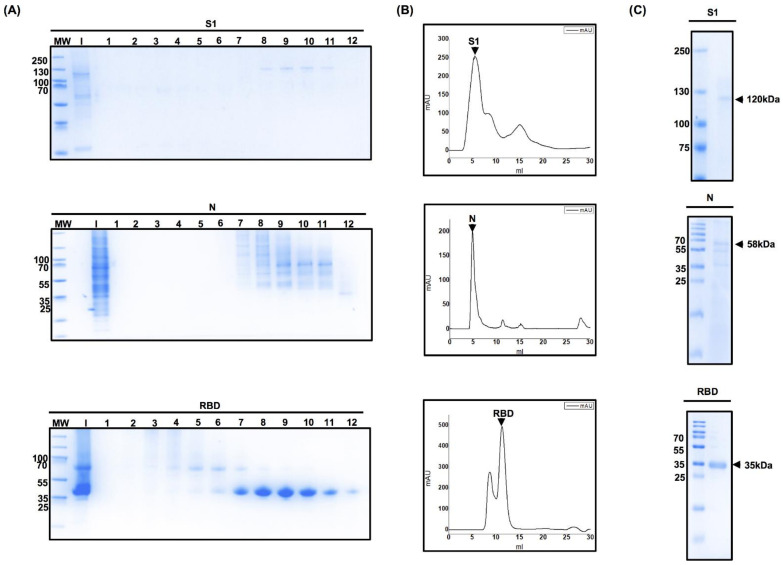
Chromatography fraction analyses of the S1 domain of spike protein, nucleocapsid, and receptor-binding domain. (**A**) Coomassie-staining SDS-PAGE analysis of protein fractions obtained using fast protein liquid chromatography. After the proteins were eluted using a 500 mM Imidazole gradient, the fractions were subjected to purification using a Superdex 75 or Superdex 200 size-exclusion column, and elution was carried out using phosphate-buffered saline. Ten to fifteen fractions of 500 µL were obtained and then analyzed by sodium dodecyl polyacrylamide gel electrophoresis. Each lane represents a different fraction eluted. (**B**) Determination of the purity of eluted protein. The absorbance of the proteins contained in the eluted fractions was measured. Measurements were carried out at 280 nm and milli-absorbance units (mAU) were plotted (*X*-axis) against the elution volume (*Y*-axis). The peaks with the highest mAU values correspond to the fractions enriched with pure protein. (**C**) These fractions were analyzed by sodium dodecyl sulfate polyacrylamide gel electrophoresis to corroborate the purity of the protein.

To produce larger quantities of recombinant proteins for subsequent assays, Expi293 cells were transfected with pcDNA3.1/S1, pcDNA3.1/N, and pCAGGS/RBD plasmids. The secretion of the recombinant protein into the culture medium is essential for the easy recovery of proteins from a cell culture system. Both S1 and N proteins contain the secretion signal sequence of the same S protein, whereas the His-tag at the C-terminal of the protein enables effective purification by affinity chromatography. We harvested the supernatant from transfected cells after centrifugation on day 5 and then filtered the sample through a 0.22 µm membrane. The filtrate was injected into a purification column. Ten to fifteen fractions of 500 µL each were obtained, analyzed by SDS-PAGE, and stained with Coomassie blue for S1, N, and RBD proteins (Figure 3A). Figure 3B shows the peaks with the highest milli-absorbance unit values, which correspond to fractions enriched with pure protein. These fractions were combined and analyzed by SDS-PAGE to corroborate the purity of the protein. SDS-PAGE analysis revealed a purity of approximately 90% (Figure 3C), and protein bands at 130 kDa, 57 kDa, and 35 kDa corresponding to S1, N, and RBD proteins obtained from Expi293 cells, respectively. We determined the protein concentration of purified proteins, which were approximately 15 mg/L of pure S1 protein, 12 mg/L of pure N protein, and 40 mg/L of pure RBD.

### 3.4. Recombinant S1 and N Are Specifically Recognized by Human Serum Samples from Patients Positive for SARS-CoV-2

**Figure 4:**
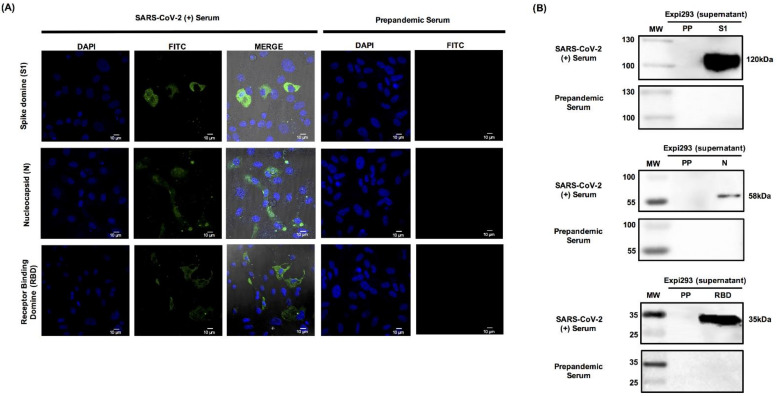
Specific recognition of the S1 domain, the nucleocapsid, and the receptor-binding domain by serum samples from patients with confirmed COVID-2019. (**A**) Expi293 cells were transfected with constructs pcDNA3.1/S1 and pcDNA3.1/N, and 24 h post-transfection, the expression of culture samples was evaluated by an immunofluorescence assay after treatment with human serum samples from patients with COVID-19, and those from pre-pandemic serum samples were evaluated as well. (**B**) The three recombinant proteins were also assessed using Western blotting. Culture samples were harvested at 24 h post-transfection and then analyzed with human serum samples from patients with confirmed COVID-19, as well as those from pre-pandemic serum. Lane 1, molecular weight marker; lane 2, eluted proteins.

Additionally, to confirm the identities of the SARS-CoV-2 proteins, we used Expi293-transfected cells with the recombinant plasmids pcDNA3.1/S1, pcDNA3.1/N, and pCAGGS/RBD. Expi293 cells transfected with the parental vector (pcDNA 3.1) were used as a negative control. At 24 h after transfection with the recombinant plasmid, cells were analyzed by immunofluorescence using serum samples from subjects with confirmed COVID-19. We observed positive immunofluorescence for S1, N, and RBD proteins, indicating that these proteins were similar to those that induce the antibodies in patients during infection. In contrast, no signal was observed when the cells transfected with the aforementioned constructs were treated with pre-pandemic sera. Furthermore, the cells transfected with the parental vector pcDNA3 did not exhibit a positive signal when treated with the sera from individuals with confirmed COVID-19 [27] (Figure 4A).

Additionally, and to confirm the identity of our recombinant proteins, the supernatants obtained from transfected cells were harvested and concentrated in 10 kDa columns (Millipore, Billerica, MA, USA), and 50 μg of each sample was resolved by 15% SDS-PAGE. Western blot analysis was performed using serum samples from patients with confirmed COVID-19. Figure 4B shows the presence of protein bands with molecular weights similar to those observed with the anti-His antibody (Figure 2) for S1, N, and RBD. However, a weaker reactivity against RBD was observed in contrast to those against S1 and N proteins. Figure 4A shows the FITC signal, with the highest yield obtained for RBD.

### 3.5. Evaluation of Antigenicity of N, S1, and RBD Proteins in a Mouse Model

**Figure 5:**
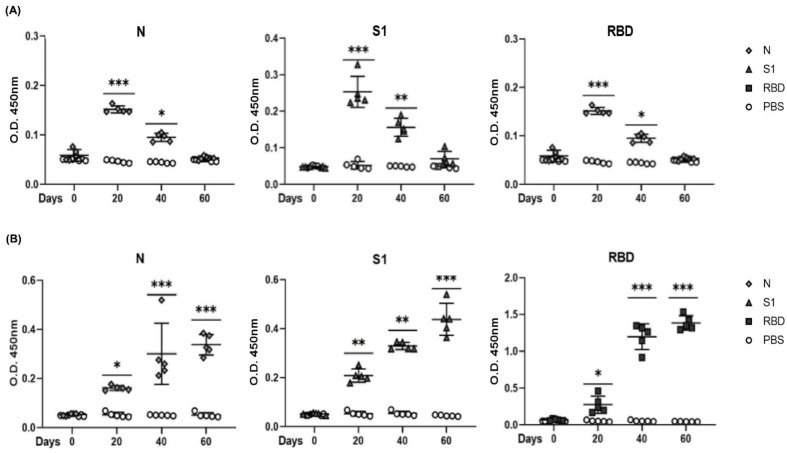
S1, N, and RBD recombinant proteins induced an antibody response in mice. Groups of five BALB/c six-week-old were immunized with 10 μg/mL three times with S1, N, and RBD proteins. Serum samples were collected at 0, 20, 40, and 60 days (one day before each immunization). Specific IgM (**A**) and IgG (**B**) antibodies were evaluated for each group. Specific antibodies were determined by ELISA, whereby S1, N, and RBD were used as antigens and horseradish peroxidase-bound goat anti-mouse IgM or IgG was used as the secondary antibody. The respective pre-immune serum samples (day 0) were analyzed as controls simultaneously, along with the group immunized with PBS. All sera were diluted to 1:100. For more than two samples, nonparametric ANOVA was used; bars represent the mean ± SD. * *p* < 0.05, ** *p* < 0.01, *** *p* < 0.001. *p* ≤ 0.05 was considered significant. Prism v.6 software was used for statistical analyses (GraphPad Inc., La Jolla, CA, USA).

We evaluated the immunogenicity of the recombinant proteins N, S1, and RBD (as positive control). Four groups of five mice each were intraperitoneally immunized with three doses of 10 µg of one of the three proteins, and the last group was immunized only with PBS. The immune response of mice was assessed at days 0, 20, 40, and 60 after immunization. The three groups inoculated with recombinant proteins showed different levels of IgM, as expected Figure 5A. The temporal induction of anti-spike IgM was faster than that of IgG. None of the four groups showed a response on day 0. Likewise, none of the three groups immunized with proteins showed a substantial IgG response after two doses. Figure 5B, Seroconversion was observed in all five mice from the three groups immunized with S1, N, and RBD. The mean value of IgG after the first immunization was 0.25 OD in all the three groups. After the second immunization, the S1- and N-immunized groups showed a mean value of 0.35 OD in contrast to the group immunized with RBD, which had a higher IgG antibody response of 1.4 OD. By day 60, the groups immunized with S1, N, and RBD proteins showed high responses, with the RBD-immunized group as the best responders. None of pre-immune serum samples or the mice immunized with PBS at the same dilution showed any response. This result confirms that the three proteins induce B cell activation and differentiation to plasmatic cells that produce specific antibodies against S1, N, and RBD proteins. Figure 5A,B.

### 3.6. Potential Use of N, S1, and RBD as Antigens in an ELISA Assay

To evaluate the performance of S1 and N as diagnostic antigens, these molecules were evaluated by enzyme-linked immunosorbent assays (ELISAs). ELISA plates were covered with 5 µg/mL of S1 and N or RBD (control), and these were incubated with serum samples collected from COVID-19-positive human sera, and pre-pandemic serum samples as controls.

**Figure 6:**
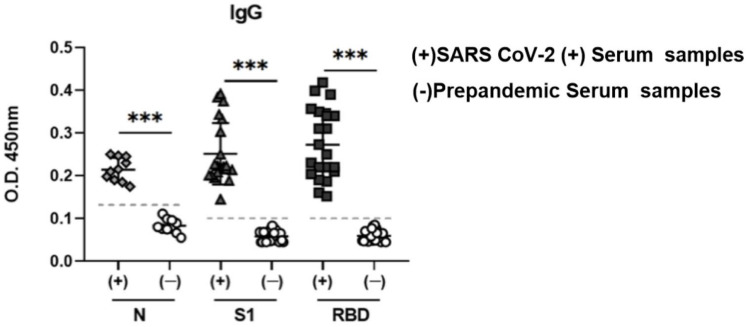
Enzyme-linked immunosorbent assay to determine recognition of the S1 domain of spike protein, nucleocapsid, and RBD by serum samples from patients with confirmed COVID-19. Ten serum samples from patients with serologically confirmed COVID-19 and 8 control serum samples were evaluated at a dilution of 1:100, and a horseradish peroxidase-conjugated rabbit anti-human IgG was used as the secondary antibody. Bars represent the mean ± SD., *** *p* < 0.001. *p* ≤ 0.05 was considered significant. Prism v.6 software was used for statistical analyses (GraphPad Inc., La Jolla, CA, USA).

Regarding the S1 antigen, the pre-pandemic control samples showed an absorbance reading of 0.05 in contrast to COVID 19-positive samples that showed an OD of 0.25 (Figure 6, middle). Similarly, COVID-19-positive samples also showed a significantly higher OD with RBD, with a median value of 0.3 OD and up to 0.42 in some samples, in contrast with very low OD in the negative control samples (Figure 6 right). A clear response was observed when the N protein was used as an antigen compared to that of the control samples, although the response against the N protein was lower than those against S1 and RBD (Figure 6, left). Importantly, the three antigens evaluated in pre-pandemic serum samples did not show a positive response in ELISA, demonstrating that these may be useful as antigens in diagnostic and seroprevalence studies.

## 4. Discussion

The COVID-19 pandemic, caused by the novel zoonotic SARS-CoV-2, is a worldwide public health concern that requires urgent attention [27,28]. The pandemic has resulted in the shortage of raw materials commonly used in laboratories. This problem has been much more severe for the supply of diagnostic reagents for COVID-19, making it imperative to develop efficient, reliable, and cost-effective strategies that help meet the manufacturing needs of each country. Thus, large amounts of high-quality viral protein components of SARS-CoV-2 are needed worldwide.

The identification and development of suitable antigens represents the first step in addressing the antibody immune response against SARS-CoV-2; thus, both T and B cell epitopes have been mapped for S, N, M, and E proteins [28,29]. The full-length S protein, organized in two domains, S1 and S2, is a fundamental protein involved in the entry of SARS-CoV-2 into host cells [13]. The S1 domain contains the RBD, which induces the production of neutralizing antibodies that prevent host cell attachment and infection [29,30]. Thus, not only is the S protein used in diagnosis and seroprevalence studies, but it can also be used to design and develop vaccines [29,30].

This study demonstrates a reliable strategy for designing and producing SARS-CoV-2 antigens, potentially aiding in their use in diagnosis and surveillance studies of the population. Several reports have described different strategies for producing recombinant antigens, such as full-length S1, RBD, or N of SARS-CoV-2 from various eukaryotic expression systems, including CHO, 293, and insect cells, and in prokaryotic cells, such as *E. coli,* for their potential production [31]. In this work, we used Expi293 cells, considering that the recombinant protein should resemble the protein synthetized in the organism as close as possible, with the same post-translational modification folding and activity state as those found in nature. These cells can provide these elements while giving a superior yield when compared to other mammalian cell expression systems [32,33]. Interest in Expi293 cells has greatly increased, as large-scale transient transfection can be performed to produce large quantities of protein in a short time [34,35].

All our target proteins were successfully overexpressed and purified. Different yields for each antigen were obtained: 15 mg/L of S1, 12 mg/L of N, and 40 mg/L of RBD. Notably, S1 and N proteins showed lower yields after cloning into pcDNA3.1, which can be explained by the different promotors used; our constructs were under control of the CMV promotor, whereas RBD was under control of the synthetic promotor CAGG, which may have led to expression differences. It is well known that the promotor is a critical element in plasmids used to express heterologous proteins. Additionally, construct sizes may influence the yield as well [36].

Additionally, pH is a factor that significantly impacts cell growth, recombinant protein production, cell metabolism, and protein glycosylation [37]. Interestingly, N and S1 were produced at slightly different pH levels, which may have impacted the yield of recombinant proteins. Another study of RBD production achieved high yields (90 mg/L of REF) using the Expi293 system; however, most studies reported values of around 20 mg/L of the RBD protein, in line with the very good yields obtained in our laboratory: 15 mg/L of S1, 12 mg/L of N, and 40 mg/L of RBD. Our lab performed transfection experiments in 250 mL culture flasks in 100 mL of medium. It is well-documented that dissolved oxygen tension in the culture medium affects productivity and cell metabolism. Thus, the aeration and distribution of CO_2_ in a smaller volume may influence yield [38,39].

Western blot analysis using anti-His antibodies revealed that the recombinant product was produced and purified with sufficient yield and purity. Furthermore, our designed proteins were clearly recognized in the sera of individuals who had recovered from COVID-19, through Western blotting, immunofluorescence, and ELISA assays. These results demonstrate the importance of these proteins in diagnostic and seroprevalence studies [34]. However, our experiments were performed using a very small number of sera samples, and additional studies of larger numbers of sera samples are needed to validate and standardize the findings of our ELISA assay.

Validated serological assays of COVID-19 are useful for determining the anti-SARS-CoV-2 response in infected individuals to understand the protection offered by the antibodies, not only in vaccinated subjects but also in future coronavirus infections. Epidemiologically, the assays can be used to identify the proportion of individuals exposed to the virus in various populations and evaluate the prevalence of asymptomatic transmission and risk factors for acquiring the disease, which is a key priority in current COVID-19-associated research [40].

Further studies should focus on developing a quantitative serological test. Using an antigen battery will be useful for reliable, large-scale serological immunological screening.

## Data Availability

All data used to support the findings presented here are available upon request to the corresponding author. The clinical data are not publicly available, due to ethical reasons related to the preservation of the privacy of volunteers.
